# Characterization of Cu_2_O/CuO heterostructure photocathode by tailoring CuO thickness for photoelectrochemical water splitting†

**DOI:** 10.1039/d1ra08863g

**Published:** 2022-01-20

**Authors:** Dasol Jeong, Woohyeon Jo, Jaebum Jeong, Taegeon Kim, Seungyeon Han, Min-Kyu Son, Hyunsung Jung

**Affiliations:** Nano Convergence Materials Center, Korea Institute of Ceramic Engineering & Technology (KICET) Jinju 52851 Republic of Korea minkyu.son@kicet.re.kr hsjung@kicet.re.kr; Department of Materials Science and Engineering, Pusan National University Busan 46241 Republic of Korea; Department of Materials Engineering, Hanyang University Ansan 15588 Republic of Korea

## Abstract

Cu_2_O/CuO heterostructure is a well-known strategy to improve the performance of Cu_2_O photocathodes for photoelectrochemical (PEC) water splitting. The CuO thickness in the Cu_2_O/CuO heterostructure is considered as a critical factor affecting the PEC performance because it is highly related to the light utilization and charge separation/transport. In this study, the Cu_2_O/CuO photocathode tailoring the CuO thickness was investigated to examine the CuO thickness influence on the PEC performance. Cu_2_O/CuO photocathodes were prepared by the electrodeposition and subsequent thermal annealing process and the Cu_2_O/CuO heterostructure was controlled by the annealing temperature and time. It was demonstrated that the increased CuO thickness enhances the light absorption in the long wavelength region and improves the charge separation by the reinforced band bending. However, the thick CuO hinders the efficient charge transport in the Cu_2_O/CuO heterostructure, resulting in the decreased PEC performance. Therefore, it is necessary to optimize the CuO thickness for the enhanced PEC performance of Cu_2_O/CuO photocathodes. Consequently, the Cu_2_O/CuO photocathode consisting of the similar CuO thickness with its minority carrier diffusion length (∼90 nm) was fabricated by annealing at 350 °C for 20 min, and it shows the optimal PEC performance (−1.2 mA cm^−2^ at 0 V *vs.* RHE) in pH 6.5 aqueous solution, resulting from the enhanced light utilization and the reinforced band bending.

## Abstract 2

Optimization of CuO thickness in the Cu_2_O/CuO photocathode by controlling the annealing time: optimal thickness of CuO induces the improved light utilization and band bending, resulting in the enhanced photoelectrochemical performance.

## Graphical abstract

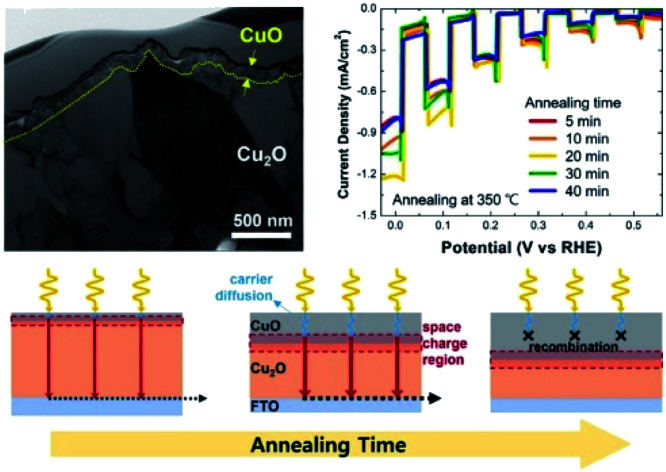

## Introduction

Cuprous oxide (Cu_2_O) is considered as a promising photocathode material for photoelectrochemical (PEC) water splitting to generate hydrogen, due to its inherent characteristics. It is a p-type semiconductor with the conduction band edge lying more negative than the hydrogen evolution reaction (HER) potential, which enables to generated hydrogen.^1–3^ In addition, it is visible light responsible due to a direct bandgap of 2.0–2.5 eV. Hence, it is theoretically possible to produce the current density of −14.7 mA cm^−2^, which corresponds to the solar-to-hydrogen conversion efficiency of 18%.^1,4–6^ Moreover, it is a non-toxic and earth abundant material with a high hall mobility of 90 cm^2^ V^−1^ s^−1^.^1,7^ Therefore, it is suitable for the efficient and low-cost PEC water splitting.

Heterostructure has been introduced to improve the PEC performance of Cu_2_O photocathodes. In general, the heterostructure Cu_2_O photocathodes with a n-type semiconductor such as TiO_2_ (ref. 8 and 9) and ZnO^10^ show the enhanced charge separation and transfer, resulting in the improved PEC performance, due to the band bending by the band alignment in the Fermi level equilibration. Especially, the heterostructure with the TiO_2_ contributes to improve the stability of Cu_2_O photocathodes, as well as its PEC performance, because TiO_2_ is an intrinsic stable oxide in the water.^8,9^ On the other hand, the heterostructure Cu_2_O photocathodes with a p-type semiconductor is also well-known.^4,11,12^ Especially, Cu_2_O/CuO heterostructure has been received lots of attention because it can be easily fabricated from the Cu_2_O by thermal annealing process. Although the CuO is a p-type semiconductor, its conduction and valence band edges are located at more positive potential than Cu_2_O. Therefore, the band bending from Cu_2_O/CuO heterostructure can suppress electron–hole recombination, resulting in the enhanced PEC performance.^2–4^ Moreover, the Cu_2_O/CuO heterostructure is able to utilize more light than Cu_2_O photocathode because the CuO has a narrow bandgap of 1.3–1.7 eV with a high absorption coefficient.^4,11–13^ Due to these advantages, many Cu_2_O/CuO heterostructures have so far been suggested, such as Cu_2_O/CuO/TiO_2_,^14^ Cu_2_O/CuO/CuWO_3_,^15^ Cu_2_O/CuO/C,^16^ Cu_2_O/CuO/CuS,^17,18^ Cu_2_O/CuO/Pt^19^ and Cu_2_O/CuO/Cu(OH)_2_.^20^ However, the Cu_2_O/CuO heterostructure has still not been optimized and further studied, despite its positive effect on the PEC performance.

The thickness of CuO in the Cu_2_O/CuO heterostructure is a critical factor affecting on the PEC performance of Cu_2_O/CuO photocathode. Thin CuO does not provide enough bend bending for improving charge separation and transfer due to the small space charge region. On the other hand, thick CuO disturbs the light absorption to the Cu_2_O layer when the light is illuminated through the front side of the photocathode. In addition, it hinders the efficient charge transport in the CuO layer because it has a short carrier diffusion length (10–200 nm).^21–23^ Therefore, it is significant to control the CuO thickness in the Cu_2_O/CuO heterostructure for the optimal PEC performance of Cu_2_O/CuO heterostructure (Cu_2_O/CuO) photocathode.

In this work, the Cu_2_O/CuO heterostructure tailoring CuO thickness was investigated to demonstrate the CuO thickness influence on the PEC performance of Cu_2_O/CuO photocathodes. The Cu_2_O/CuO photocathode was fabricated by electrodeposition and subsequent thermal annealing process. The Cu_2_O/CuO heterostructure was controlled by the thermal annealing temperature and time because these fabrication parameters are directly related to the formation of CuO from the surface of Cu_2_O film. As a result, the intact Cu_2_O/CuO heterostructure was obtained by the thermal annealing at 350 °C, while the Cu_2_O/CuO photocathode annealed at this temperature for 20 min showed the best PEC performance. Based on the optical analysis and Mott–Schottky measurement, it was demonstrated that the thickness of CuO fabricated by these fabrication conditions is optimal for the efficient charge separation/transport and light utilization, resulting in the improved PEC performance of Cu_2_O/CuO photocathode.

## Experimental details

The fluorine doped tin oxide (FTO, 7 Ω sq^−1^, Sigma Aldrich) substrate was cleaned by ultra-sonication using acetone and ethanol for 5 min, respectively, before the Cu_2_O deposition. The Cu_2_O films were deposited on the cleaned FTO substrate by the electrodeposition. In details, the copper(ii) lactate aqueous solution (pH 12) containing 0.06 M CuSO_4_ (YAKURI, Japan), 1 M lactic acid (DAEJUNG, Korea), 0.16 M K_2_HPO_4_ (DAEJUNG, Korea), and 1.26 M KOH (DAEJUNG, Korea) was used as an electrolyte for the electrodeposition. The bath temperature was maintained at 30 °C using jacketed beaker with water circulation system during the electrodeposition. The electrodeposition was carried out in a standard three-electrode system with the FTO as a working electrode, a saturated calomel electrode (SCE) reference electrode and a platinum (Pt) mesh counter electrode (30 × 30 mm^2^) at a constant voltage of −0.6 V (*vs.* SCE) applied by a potentiostat (PMC-1000, AMETEK). The deposition area was fixed at 2.54 cm^2^ by a home-made Teflon holder. The deposition was implemented until an average charge of 2.06 C was passed. It expects the thickness of fabricated Cu_2_O film is approximately 1 μm, which is derived by Faraday's law based on the passed charge, deposition area, density and molar ratio of Cu_2_O.^24^ To form Cu_2_O/CuO heterostructure, the electrodeposited Cu_2_O film was annealed at different temperatures in air. In addition, the thickness of CuO was controlled by the thermal annealing time at the temperature to complete the Cu_2_O/CuO heterostructure.

To characterize pristine and annealed Cu_2_O samples, the morphological analysis was carried out using a field emission scanning electron microscopy (FE-SEM, D8 Advance, Bruker) and a transmission electron microscopy (TEM) obtained in Titan Themis Z (FEI) with an accelerating voltage of 300 kV. The structural and compositional analysis was conducted using X-ray diffraction (XRD) (JSM-6700F, JEOL) with Cu Kα radiation (*λ* = 0.1518 nm). In addition, the X-ray photoelectron spectroscopy (XPS) measurement was carried out using a XPS system with automated surface analysis (NEXSA, Thermo Fisher Scientific) to further characterize the fabricated electrode. The optical properties were characterized using UV-Vis spectrophotometer (V-670, JASCO) to determine the absorption coefficients and the band gap.

Electrochemical experiments were carried out in the three-electrode system consisting of the SCE reference electrode and the 10 × 10 mm^2^ Pt mesh counter electrode. Measured data were acquired by the potentiostat (PMC-1000, AMETEK). The PEC performance of Cu_2_O/CuO photocathode was measured in a 0.1 M Na_2_SO_4_ electrolyte (pH 6.25) under chopped illumination (1 sun, 100 mW cm^−2^) from a solar simulator (TLS-300XU, Newport). The area of photocathodes was 0.384 cm^2^ controlled by the commercially available Teflon cell (WizMAC, Korea). The measured potential (*vs.* SCE) was converted into the reversible hydrogen electrode (RHE) scale. The Mott–Schottky measurement was carried out in a 0.1 M Na_2_SO_4_ electrolyte (pH 6.25) to derive the flat band potential and energy band level information. It was performed at a frequency of 1 kHz in the range of potential between 0 V and 1.4 V *vs.* RHE.

## Results and discussion

**Fig. 1:**
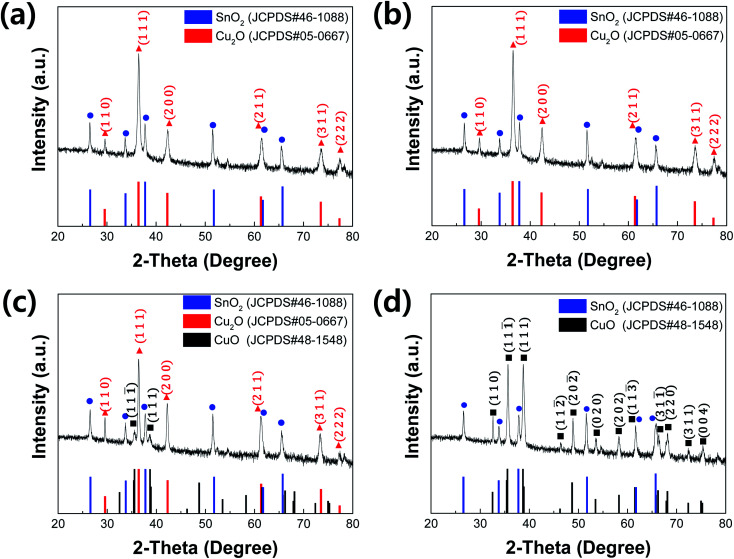
XRD patterns of electrodeposited Cu_2_O films (a) before annealing and (b–d) after annealing at 150 °C, 350 °C, 550 °C for 20 min, respectively.

The electrodeposited Cu_2_O films were annealed to fabricate Cu_2_O/CuO photocathode because the surface of Cu_2_O is easily oxidized into CuO by the annealing in air. Fig. 1 and Table 1 show the XRD patterns and peak analyses results of electrodeposited Cu_2_O films before and after thermal annealing. All films showed XRD peaks indexed to the tin oxide (SnO_2_, JCPDS No. 46-1088) because they were fabricated on the FTO substrate. The crystallite size of films was calculated using the Scherrer's equation.^25^
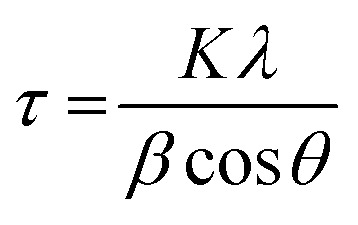
where *K* = 0.94 is the shape factor, *λ* = 0.15418 is the wavelength of the X-ray, *β* is full width at half maximum (FWHM), and *θ* is the Bragg angle. As shown in Fig. 1a, the film before annealing was indexed as cuprite Cu_2_O (JCPDS No. 05-0667) without any impurity peaks. It means that the pristine electrodeposited film is pure Cu_2_O. On the other hand, no other peaks were still observed except ones indexed to the Cu_2_O with the dominant orientation of (1 1 1) in the film after annealing at 150 °C for 20 min. It indicates that the annealing at this temperature is not sufficient to form CuO because the transformation of Cu_2_O into CuO were generally occurred by thermal oxidation at the temperature over 200 °C in air.^26^ Interestingly, only crystallite size of film was increased from 36.7 nm to 39.4 nm, as shown in Table 1. It demonstrates that the mild annealing at 150 °C leads to the improved crystallite size of Cu_2_O, not the transformation into CuO.

**Table 1:** XRD peak analyses of electrodeposited Cu_2_O films before and after thermal annealing

Sample	Peak	Orientation	2-Theta	FWHM	Crystallite size
Pristine Cu_2_O	Cu_2_O (JCPDS#05-0667)	(1 1 1)	36.4	0.238	36.7
Cu_2_O-150 °C	Cu_2_O (JCPDS#05-0667)	(1 1 1)	36.5	0.222	39.4
Cu_2_O-350 °C	Cu_2_O (JCPDS#05-0667)	(1 1 1)	36.3	0.178	49.1
	CuO (JCPDS#48-1548)	(1 1–1)	35.6	0.279	31.3
Cu_2_O-550 °C	CuO (JCPDS#48-1548)	(1 1–1)	35.6	0.188	46.4

However, although the XRD peaks indexed to Cu_2_O are dominant, XRD peaks indexed to tenorite CuO (JCPDS No. 48-1548) were observed in the film after annealing at 350 °C for 20 min, as shown in Fig. 1c. It supports the existence of Cu_2_O and CuO in the film, resulting from the oxidization of Cu_2_O surface due to the sufficient annealing temperature. In addition, the crystallite size of Cu_2_O (49.1 nm) became larger than one of the annealed film at 150 °C, indicating that thermal annealing process at the high temperature contributes to improve the crystallite size. On the other hand, only XRD peaks indexed to CuO were observed in the film after annealing at 550 °C for 20 min, as shown in Fig. 1d. It means that the electrodeposited Cu_2_O is entirely oxidized into the pure CuO with the dominant orientations of (1 1 ¯1) and (1 1 1) due to such high temperature annealing condition.

**Fig. 2:**
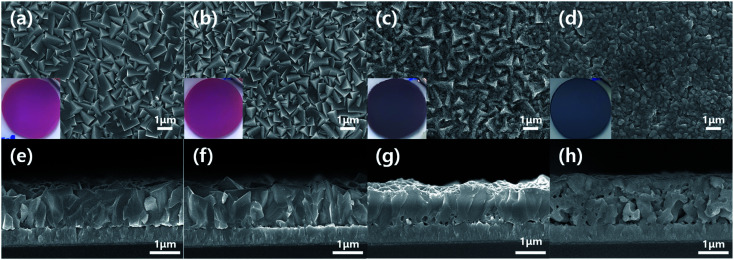
Top and cross-sectional SEM images of electrodeposited Cu_2_O films (a and e) before annealing and after annealing at (b and f) 150 °C, (c and g) 350 °C and (d and f) 550 °C for 20 min.

Fig. 2 shows SEM images and pictures (insets of Fig. 2a–d) of electrodeposited Cu_2_O films before and after annealing at different temperatures. The film before annealing showed the inherent morphological characteristics of electrodeposited Cu_2_O:^11,27^ its color is red, while its surface is polyhedral (Fig. 2a). It is conclusive evidence that the electrodeposited film is the pure Cu_2_O. Its thickness is approximately 1.1 μm, which is well matched to one designed based on Faraday's law. These morphological characteristics were still maintained after annealing at 150 °C for 20 min because the Cu_2_O film was not oxidized into CuO by such mild annealing process (Fig. 2b). On the other hand, the surface of Cu_2_O film was entirely changed into the granular morphology after annealing at 550 °C for 20 min (Fig. 2d). Furthermore, the color of film was turned into black, which is an inherent color of CuO (inset of Fig. 2d). Its thickness became thicker than one of electrodeposited Cu_2_O because the Cu_2_O is expanded and recrystallized into CuO by the thermal annealing at the high temperature. These are in good agreement with the XRD results.

**Fig. 3:**
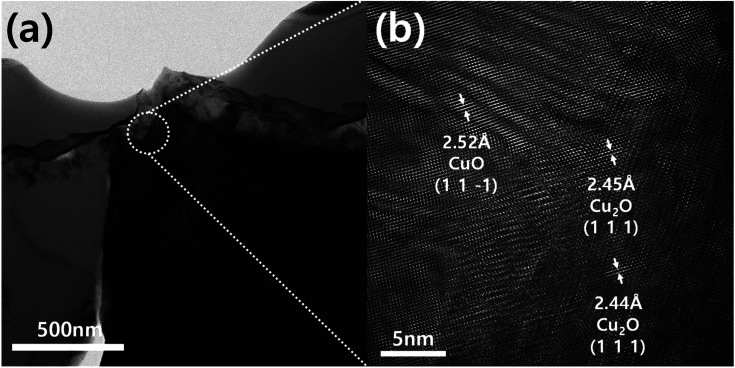
TEM analysis of the annealed Cu_2_O film at 350 °C for 20 min: (a) low magnification image and (b) high resolution image showing the Cu_2_O/CuO heterostructure.

Meanwhile, the Cu_2_O film annealed at 350 °C for 20 min showed the dual morphological characteristics of Cu_2_O and CuO, as illustrated in Fig. 2c. The color of film was turned into the reddish black, which is a mixture color of Cu_2_O (red) and CuO (black). In addition, the granular morphology was found on the surface of the film, even though the surface was still polyhedral. Thus, it is likely to be a Cu_2_O film covered CuO thin film. In other words, the annealing at 350 °C for 20 min makes the electrodeposited pure Cu_2_O film into the Cu_2_O/CuO heterostructure film. The exact Cu_2_O/CuO heterostructure was revealed by the TEM analysis. Fig. 3 shows TEM images of the annealed Cu_2_O film at 350 °C for 20 min at low magnification and high resolution. The boundary interface between two different layers was found in the Fig. 3a. In addition, the different crystal lattice spacings were observed in the boundary interface (Fig. 3b): the value of 2.52 Å in the overlayer corresponds to (1 1 ¯1) lattice plane of CuO (tenorite, JCPDS No. 48-1548), while one of 2.44–2.45 Å in the bottom layer corresponds to (1 1 1) lattice plane of Cu_2_O (cuprite, JCPDS No. 05-0667). It demonstrates that the mixed film with Cu_2_O and CuO is a Cu_2_O/CuO heterostructure film. The CuO layer was homogeneously deposited on the Cu_2_O with a thickness of 90 ± 15 nm (Fig. S1†). Based on these observations, it is concluded that the optimal annealing temperature is 350 °C to form the intact Cu_2_O/CuO heterostructure from the electrodeposited Cu_2_O film by the thermal annealing process.

**Fig. 4:**
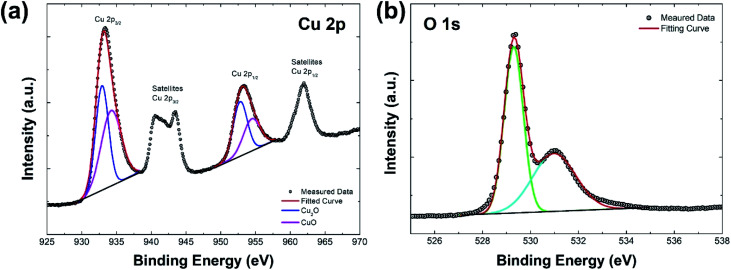
XPS spectra of the annealed Cu_2_O film at 350 °C for 20 min (a) Cu 2p and (b) O 1s.

To further confirm the characteristics of Cu_2_O/CuO heterostructure, XPS analysis of the annealed Cu_2_O film at 350 °C for 20 min was carried out, as illustrated in Fig. 4. In the XPS spectrum of Cu 2p (Fig. 4a), the peak of Cu 2p_3/2_ located at 933.4 eV and the peak of Cu 2p_1/2_ located at 953.4 eV are observed, respectively. These two peaks represent characteristics of Cu_2_O and CuO: the peaks located at 932.9 eV and 952.7 eV are related to Cu_2_O (blue lines in fitted curves), while the peaks located at 934.2 eV and 954.4 eV are attributed to CuO (pink lines in fitted curves).^28,29^ In addition, the satellite peaks of Cu 2p_3/2_ and Cu 2p_1/2_ supports the presence of CuO in the film.^29,30^ On the other hand, the dominant peak at 529.4 eV corresponds to the metal–oxygen bonds, while the peak at 531 eV is attributed to the surface hydroxide in the XPS spectrum of O 1s (Fig. 4b).^31^ These XPS results indicate that the annealed Cu_2_O film at 350 °C for 20 min is a well-structured Cu_2_O/CuO film.

**Fig. 5:**
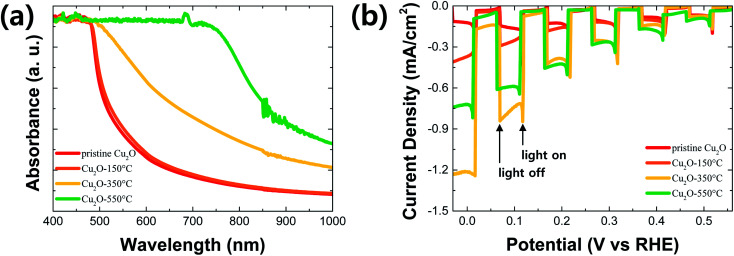
(a) Absorption spectra and (b) PEC performances in pH 6.5 aqueous solution under chopped one sun illumination: pristine Cu_2_O and annealed Cu_2_O at different temperatures for 20 min.

Fig. 5a shows absorption spectra of pristine (no annealing) and annealed Cu_2_O films at different temperatures. The absorbance of the annealed Cu_2_O film at 150 °C was similar to one of the pristine Cu_2_O because the composition of the film is not changed by the thermal annealing at this temperature. However, the absorbance in the longwave length region was gradually improved along with the increased annealing temperature above 350 °C. It is mainly caused by the increment of CuO thickness in the Cu_2_O film. In general, the band gap of CuO is smaller than one of Cu_2_O, resulting in the enhanced light utilization in the longwave length region.^4,11,12^ To confirm the band gap of Cu_2_O and CuO in the Cu_2_O/CuO heterostructure, Tauc plots (Fig. S2†) were drawn using absorption spectra of the pristine Cu_2_O (pure Cu_2_O) and the annealed Cu_2_O at 550 °C (pure CuO), respectively. The band gap was derived by the following equation.(*αhν*)^*n*^ = *A*(*hν* − *E*_g_)where *α* is the absorption coefficient, *h* = 4.1357 × 10^−15^ eV *s* is Planck constant, *ν* is frequency, *A* is a constant, *E*_g_ is the optical bandgap energy, and *n* = 2 is a constant for direct transition.^4^ As a result, the bandgap energy of Cu_2_O is ∼2.4 eV, while CuO is ∼1.4 eV. These are in good agreement with the reported ones of Cu_2_O and CuO in the literature.^2–4^

Fig. 5b shows PEC performances of the pristine and annealed Cu_2_O photocathode in pH 6.5 aqueous solution under chopped one sun light illumination. The PEC performance of Cu_2_O photocathodes was improved after annealing at 150 °C for 20 min, even though the compositional and optical characteristics were not changed. It is likely due to the increased crystallite size of Cu_2_O, resulting in the enhanced charge transport in the film by the reduced grain boundaries. The PEC performance was further improved after annealing at 350 °C for 20 min. The photocurrent density was reached up to −1.21 mA cm^−2^ at 0 V *vs.* RHE. It is mainly caused by two factors: one is the improved light absorption in the longwave length region by the CuO overlayer, the other is the enhanced charge separation and transfer by the Cu_2_O/CuO heterostructure. The former is demonstrated by the absorption spectra of the film (Fig. 5a), while the letter is illustrated by the energy band diagram in the next paragraph in details. On the other hand, the PEC performance was decreased again after annealing at 550 °C for 20 min, even though the light absorption was more improved in the longwave length region. The annealed Cu_2_O photocathode at this temperature loses its Cu_2_O characteristics and Cu_2_O/CuO heterostructure because the entire Cu_2_O film is converted into the pure CuO film.

**Fig. 6:**
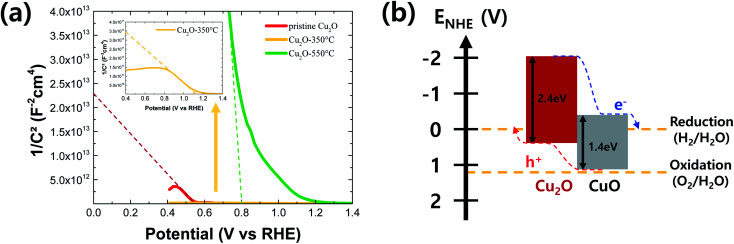
(a) Mott–Schottky plot of pristine and annealed Cu_2_O films at the different temperatures and (b) a schematic of the energy band diagram of the Cu_2_O/CuO heterostructure.

To examine the energy band diagram of Cu_2_O/CuO heterostructure, the flat band potential was estimated by the Mott–Schottky plot based on the following equation.
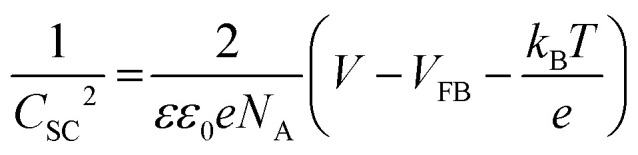
where *C*_SC_ is space-charge capacitance, *N*_A_ is the charge carrier density, *ε* is relative dielectric constant of the semiconductor (for Cu_2_O and CuO is 7.60 and 10.26, respectively^32,33^), *ε*_0_ = 8.854 × 10^−14^ F cm^−1^ is dielectric constant in free space, *e* = 1.602 × 10^−19^ is charge of electron, *k*_B_ = 1.381 × 10^−23^ is Boltzmann constant, *T* = 293 K is temperature, *V* is applied potential and *V*_FB_ is flat band potential of the semiconductor, respectively.^34^Fig. 6a shows the Mott–Schottky plots of pristine and annealed Cu_2_O films at the different temperatures. All films show negative slopes, which means that they are based on the p-type semiconductors (CuO and Cu_2_O). The flat band potential can be estimated from the extrapolated line to the *x*-axis in the Mott–Schottky plot. Based on this estimation, the pristine Cu_2_O film has an estimated flat band potential of 0.55 V *vs.* RHE, while the annealed Cu_2_O film at 550 °C (the CuO film) has an estimated flat band potential of 0.79 V *vs.* RHE. Meanwhile, the estimated flat band potential of the annealed Cu_2_O film at 350 °C (the Cu_2_O/CuO film) is 1.13 *vs.* RHE. It is greatly larger than ones of pure CuO and Cu_2_O films, indicating that the degree of band bending in the Cu_2_O/CuO heterostructure is higher than ones of CuO or Cu_2_O monostructure. It causes the efficient charge separation and transport, resulting in the improved PEC performance.^35^

Fig. 6b shows a rough energy band diagram of the Cu_2_O/CuO heterostructure using the optical band gap information (Fig. 5a) and the estimated valence band edge from the flat band potential information (Fig. 6a). The valence band edge can be estimated as the Fermi level because it is closely located at the Fermi level in the p-type semiconductor. In addition, the Fermi level is equal to the flat band potential when the light was illuminated to the semiconductor/water interface.^4^ As shown the energy levels of Cu_2_O and CuO in Fig. 6b, the electron can be efficiently moved to the semiconductor/water interface, reducing the water into the hydrogen, while the hole can be efficiently moved to the counter electrode *via* FTO substrate, oxidizing the water into the oxygen. Consequently, it was demonstrated that the Cu_2_O/CuO heterostructure is a promising strategy to improve the PEC performance of Cu_2_O photocathode by the enhanced charge separation and transport from the strong band bending effect of heterostructure.

**Fig. 7:**
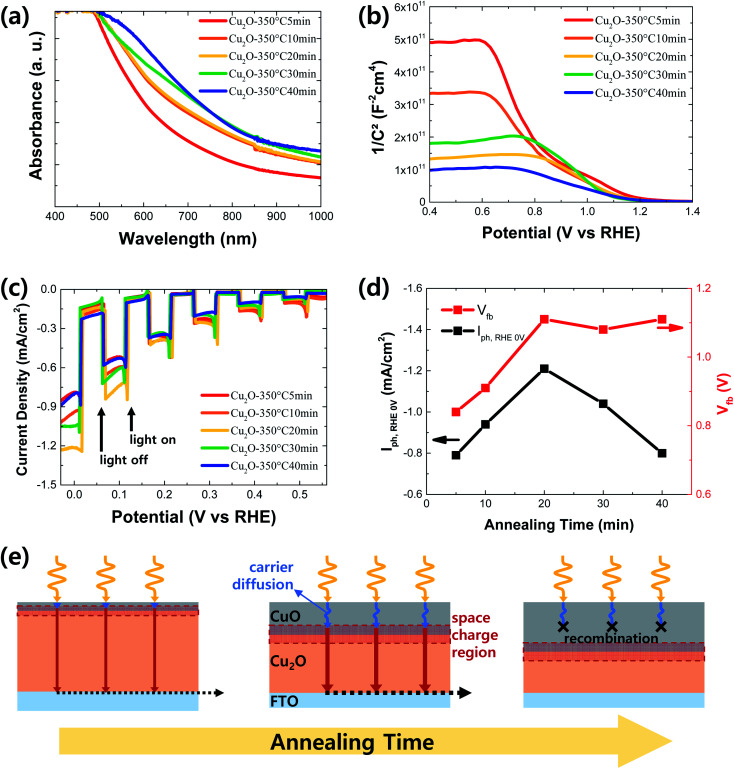
(a) Absorption spectra, (b) Mott–Schottky plots and (c) PEC performances of Cu_2_O/CuO films annealed at 350 °C for 5, 10, 20, 30 and 40 min (d) photocurrent densities at 0 V *vs.* RHE (*I*_ph, RHE 0V_) and flat band potential (*V*_fb_) according to the annealing time (all potential scales are RHE). The PEC performance was measured in pH 6.5 aqueous solution under chopped one sun illumination. (e) Schematic of Cu_2_O/CuO heterostructure with the different CuO thicknesses.

To optimize the thickness of CuO in the Cu_2_O/CuO heterostructure, it was controlled by adjusting the annealing time at the annealing temperature of 350 °C: 5, 10, 20, 30 and 40 min. As shown in Fig. S3,† CuO particles are observed on the surface of the annealed sample for 5 min. It means that the intact CuO film was not formed on the Cu_2_O film due to the short annealing time. Hence, it is difficult to determine the thickness of CuO in this sample. On the other hand, the thickness of CuO in the annealed samples for an annealing time above 10 min was gradually increased in accordance with the prolonged annealing time (Fig. S1 and S3†) because the extended annealing time provides more chances to react the Cu_2_O with the air. As shown in Fig. 7a, the light absorbance was slightly improved in the long wavelength region as the annealing time was increased. It is mainly caused by the increased thickness of CuO with a small band gap (∼1.4 eV). On the other hand, the Mott–Schottky measurement was carried out to evaluate the band bending effect of CuO thickness in the Cu_2_O/CuO heterostructure. Based on the extrapolation method using the Mott–Schottky plots of the annealed Cu_2_O film at 350 °C for different annealing time (Fig. 7b), the derived flat band potentials of the annealed Cu_2_O film at 350 °C for 5, 10, 20, 30, and 40 min are 0.84, 0.91, 1.11, 1.08, and 1.11 V *vs.* RHE, respectively. It is gradually increased along with the prolonged annealing time up to 20 min, while it is saturated around the value of 1.1 V *vs.* RHE over the annealing time of 20 min. In other words, the band bending is strengthened by increasing the CuO thickness up to approximately 90 nm, while it is almost same when the CuO thickness is over 90 nm. These optical characteristics and flat band potential information give the overall insight into the influence of CuO thickness on the PEC performance of Cu_2_O/CuO photocathodes.

Fig. 7c shows the PEC performance of Cu_2_O/CuO photocathodes with different CuO thicknesses fabricated at 350 °C for different annealing time in pH 6.5 aqueous solution under chopped one sun light illumination. In addition, the derived flat band potentials from Mott–Schottky plots and the photocurrent densities at 0 V *vs.* RHE of Cu_2_O/CuO photocathodes fabricated at 350 °C for different annealing time were summarized in Fig. 7d. The PEC performance, especially the photocurrent density, was gradually improved by increasing the annealing time up to 20 min because the light absorbance (Fig. 7a) and band bending (Fig. 7d) are enhanced in accordance with the increased CuO thickness. However, although the light absorbance was further improved and the band bending was still strong over the annealing time of 20 min, the photocurrent density was reduced, resulting in the decreased PEC performance. As a result, the Cu_2_O/CuO photocathode showed the best PEC performance by annealing the electrodeposited Cu_2_O at 350 °C for 20 min.

This can be explained in the schematic of Cu_2_O/CuO heterostructure with the different CuO thickness, as shown in Fig. 7e, in details. In the short annealing time, a thin CuO layer restricts the space charge region limiting the band bending. Hence, the flat band potential and PEC performance are low. On the other hand, the annealing time of 20 min produces the optimal CuO thickness enough to fully spread the space charge region out, showing the enhanced band bending. In addition, the CuO thickness (90 nm) in this case coincides with the minority carrier diffusion length of CuO (∼100 nm),^21–23^ resulting in the efficient charge transport. However, in the long annealing time over 20 min, the CuO thickness is much thicker than the minority carrier diffusion length of CuO. Thus, the PEC performance was reduced by the charge losses in the CuO film, although the improved band bending by the Cu_2_O/CuO heterostructure is maintained due to the sufficient space charge region. Therefore, it can be concluded that CuO thickness is a significant parameter to determine the PEC performance of Cu_2_O/CuO photocathode. Finally, it was demonstrated that the optimal CuO thickness is approximately 90 nm for the enhanced PEC performance of Cu_2_O/CuO photocathode and it can be achieved by the thermal annealing process at 350 °C for 20 min after the electrodeposition of Cu_2_O film.

## Conclusion

In this work, the Cu_2_O/CuO heterostructure was controlled by the thermal annealing temperature and time after electrodepositing Cu_2_O film to investigate the CuO thickness influence on the PEC performance of Cu_2_O/CuO photocathodes. It was found that the annealing temperature determines the formation of Cu_2_O/CuO heterostructure and the annealing time precisely controls the CuO thickness in the Cu_2_O/CuO heterostructure. The complete Cu_2_O/CuO heterostructure was formed by the annealing at 350 °C, while the CuO thickness was gradually increased by extending the annealing time. The light absorption in the long wavelength region was enhanced by increasing the CuO thickness due to the small band gap of CuO. In addition, the band bending for improving the charge separation was enhanced up to the CuO thickness of 90 nm because the increased CuO thickness provides the sufficient room for the space charge region. However, it was not further enhanced in the CuO thickness above 90 nm. Instead of this, the charge loss is likely to be increased because such thickness is thicker than the minority carrier diffusion length of CuO. As a result, the Cu_2_O/CuO photocathode with the approximate 90 nm thick CuO fabricated by annealing the electrodeposited Cu_2_O film at 350 °C for 20 min shows the best PEC performance (the photocurrent density of −1.2 mA cm^−2^ at 0 V *vs.* RHE) in pH 6.5 aqueous solution. Therefore, it was concluded that the CuO thickness should be considered for the improved PEC performance of Cu_2_O/CuO photocathodes and the similar CuO thickness with its minority carrier diffusion length in the Cu_2_O/CuO heterostructure ensures the optimal PEC performance, resulting from the improved light utilization and the enhanced band bending effect. Although the PEC performance was not comparable to other Cu_2_O/CuO photocathodes in the previous literature due to the absence of HER catalysts (Table S1†), this finding is a significant footprint for optimizing CuO thickness in the Cu_2_O/CuO photocathodes. Introduction of interlayers and the efficient HER catalysts with low cost materials would be a next step toward the further improvement of PEC performance of Cu_2_O/CuO photocathodes in the future.

## Conflicts of interest

There are no conflicts to declare.

## Supplementary Material

RA-012-D1RA08863G-s001
