# Supplementary material for: Characterization of Cu2O/CuO heterostructure photocathode by tailoring CuO thickness for photoelectrochemical water splitting
Source: RSC Adv. 2022 Jan 20;12(5):2632–40. doi: 10.1039/d1ra08863g (PMC8979049; doi:10.1039/d1ra08863g)
Supplement: RA-012-D1RA08863G-s001 [file RA-012-D1RA08863G-s001.pdf]

Electronic Supplementary Material (ESI) for RSC Advances.
This journal is © The Royal Society of Chemistry 2022

Supporting Information

Characterization of Cu_2O/CuO Heterostructure Photocathode by Tailoring CuO Thickness for Photoelectrochemical Water Splitting

Dasol Jeong[1], Woohyeon Jo[1,2], Jaebum Jeong[1], Taegeon Kim[1,3], Seungyeon Han[1,2], Min-Kyu Son[1*], Hyunsung Jung[1*]

[1] Nano Convergence Materials Center, Korea Institute of Ceramic Engineering & Technology (KICET), Jinju 52851, Republic of Korea

[2] Department of Materials Science and Engineering, Pusan National University, Busan 46241, Republic of Korea

[3] Department of Materials Engineering, Hanyang University, Ansan 15588, Republic of Korea

*E-mail: minkyu.son@kicet.re.kr ; hsjung@kicet.re.kr

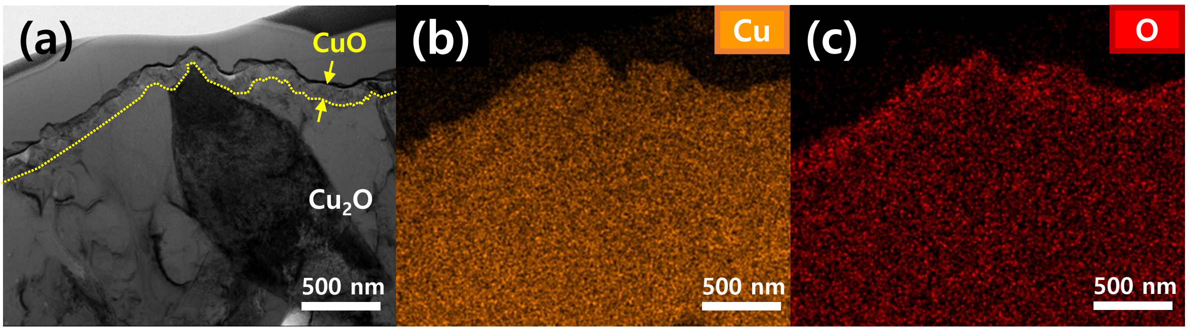

Figure S1. TEM and element mapping analysis of the annealed Cu_2O film at 350 °C for 20 min.

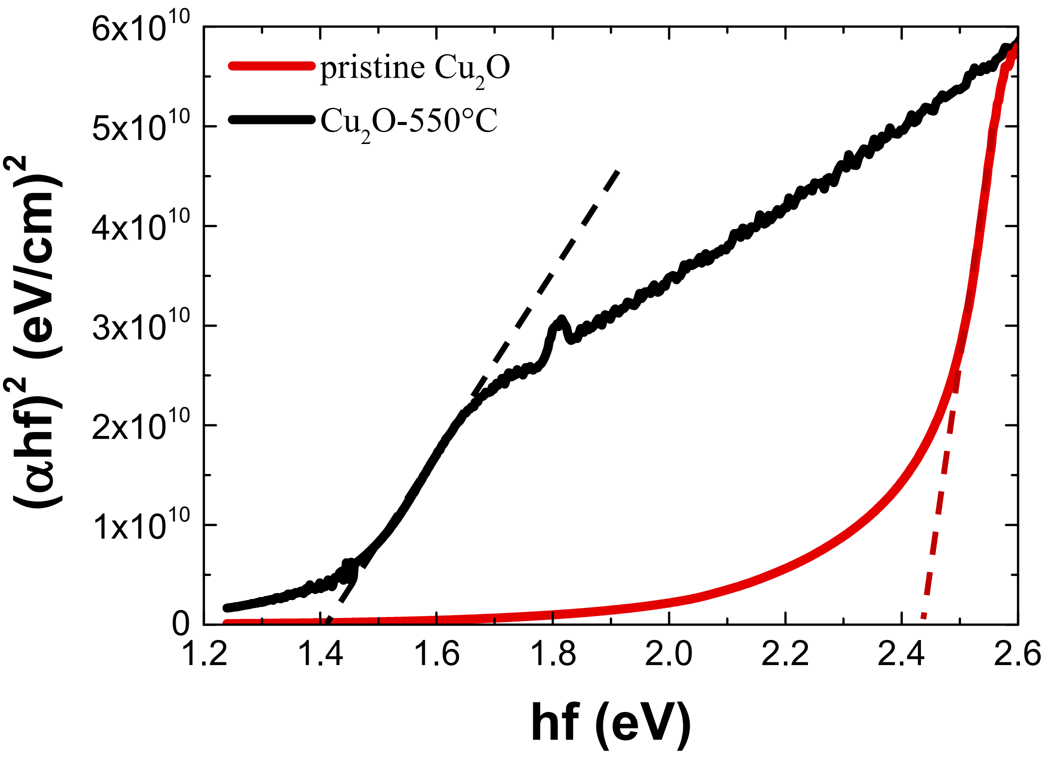

Figure S2. Tauc plot of pristine Cu_2O (pure Cu_2O) and annealed Cu_2O (pure CuO) at 550 °C for 20 min.

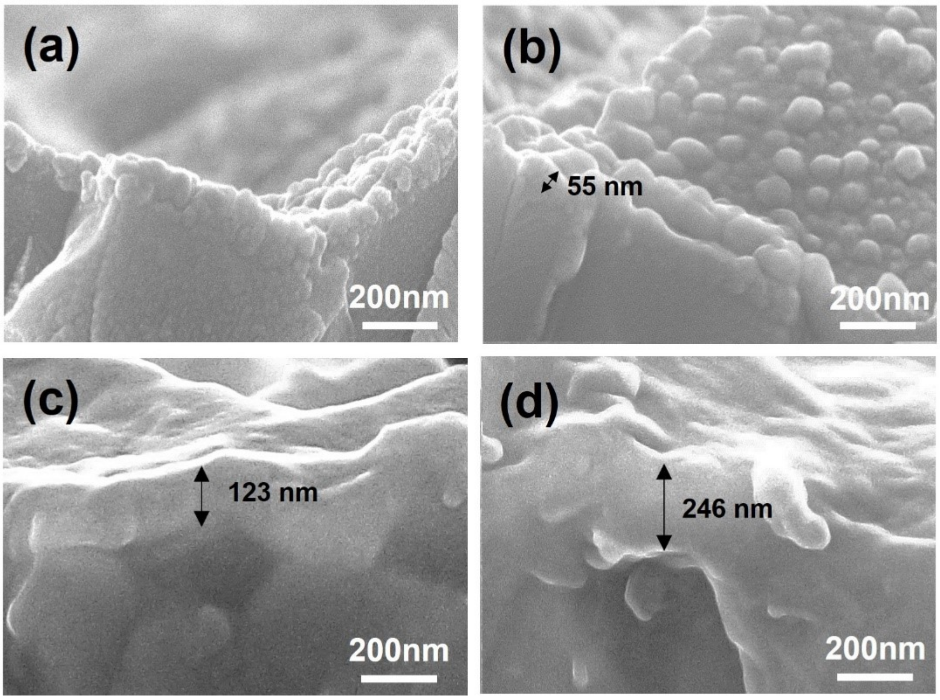

Figure S3. Cross-sectional SEM images of Cu_2O/CuO films annealed at 350 °C for (a) 5 min, (b) 10 min, (c) 30 min, (d) 40 min.

Table S1. PEC performances of Cu_2O/CuO photocathodes in our work and other literatures.

Photocathode	Electrolyte	Current density (mA/cm^2)	Reference
Cu_2O/CuO	0.5 M Na_2SO_4 (pH 6)	- 2.8 (0 V vs RHE)	[4]
Cu_2O/CuO	1 M Na_2SO_4	- 1.4 (0 V vs RHE)	[11]
Cu_2O/CuO	0.5 M Na_2SO_4 (pH 6)	- 0.45 (0.25 V vs RHE)	[12]
Cu_2O nanowire/CuO nanoflake	0.5 M Na_2SO_4 (pH 6)	- 4 (0V vs RHE)	[13]
Cu_2O nanowire/CuO/TiO_2	1 M Na_2SO_4 (pH 5)	- 0.87 (0 V vs RHE)	[14]
$Cu_2O/CuO/CuWO_3$	1 M Na_2SO_4 (pH 7)	- 1.9 (0 V vs RHE)	[15]
$Cu_2O/CuO/C$	1 M Na_2SO_4 (pH 5.5)	- 6.5 (0 V vs RHE)	[16]
$Cu_2O/CuO/CuS/Pt$	1 M Na_2SO_4 (pH 5)	- 6 (0V vs RHE)	[17]
$Cu_2O/CuO/CuS$	0.5 M Na_2SO_4 (pH 5)	- 1.38 (0V vs RHE)	[18]
$Cu_2O/CuO/Pt$	·	- 1.99 (0V vs RHE)	[19]
$Cu_2O/CuO/Cu(OH)_2$	0.5 M Na_2SO_4 (pH 6.8)	- 2.3 (0V vs RHE)	[20]
Cu_2O/CuO	0.1 M Na_2SO_4 (pH 6.25)	- 1.2 (0V vs RHE)	Our work
